# Circular RNA Expression Profiles and Bioinformatic Analysis in Mouse Models of Obstructive Sleep Apnea-Induced Cardiac Injury: Novel Insights Into Pathogenesis

**DOI:** 10.3389/fcell.2021.767283

**Published:** 2021-11-08

**Authors:** Suxian Lai, Lijun Chen, Pingyun Zhan, Guofu Lin, Hai Lin, Huibin Huang, Qingshi Chen

**Affiliations:** ^1^ Department of Neonatology, The First Hospital of Quanzhou Affiliated to Fujian Medical University, Quanzhou, China; ^2^ Department of Endocrinology and Metabolism, The Second Affiliated Hospital of Fujian Medical University, Quanzhou, China; ^3^ Department of Cardiology, Haidu Hospital, Quanzhou, China; ^4^ Department of Respiratory and Critical Care Medicine, The Second Affiliated Hospital of Fujian Medical University, Quanzhou, China

**Keywords:** CircRNAs, obstructive sleep apnea, cardiac injury, chronic intermittent hypoxia, expression profile

## Abstract

Circular RNAs (circRNAs) participate in the development of various kinds of diseases. However, the function and roles of circRNAs in obstructive sleep apnea (OSA)-induced cardiovascular disease remain poorly understood. Therefore, we sought to explore the circRNA expression profiles and predict their functions in OSA-induced cardiac injury with the use of bioinformatics analysis. The model of OSA was established in mouse treated by chronic intermittent hypoxia (CIH) exposure. Then, we screened the circRNA profile using circRNA microarray. By comparing circRNA expression in three matched pairs of CIH-treated cardiac tissues and controls, differentially expressed circRNAs were identified in the CIH groups. Comparison of the selected circRNAs expression levels was performed between qRT-PCR and microarray. Meanwhile, we employed Gene Ontology (GO) and Kyoto Encyclopedia of Genes and Genomes (KEGG) pathway analyses to predict the functions of these selected circRNAs. Finally, we constructed a circRNA-miRNA-mRNA network based on the target prediction. It was found that a total of 124 circRNAs were differentially expressed in CIH-treated cardiac tissues (*p* ≤ 0.05, fold-change ≥ 1.5). Among them, 23 circRNAs were significantly down-regulated, and the other 101 were up-regulated. Then, ten circRNAs were randomly selected to validate the reliability of the microarray results by using qRT-PCR. Next, we conducted the GO and KEGG pathway analysis to explore the parental genes functions of differentially expressed circRNA. Finally, two significantly differentially expressed circRNAs (mmu_circRNA_014309 and mmu_circRNA_21856) were further selected to create a circRNA-miRNA-mRNA regulation network. Our study did first reveal that the differentially expressed circRNAs played a vital role in the pathogenesis of OSA-induced cardiac damage. Thus, our findings bring us closer to unraveling the pathophysiologic mechanisms and eliciting novel therapeutic targets for the treatment of OSA-associated cardiovascular diseases.

## Introduction

Obstructive sleep apnea (OSA) is a complex, multifactorial disorder defined by chronic intermittent hypoxia (CIH), which affected millions of people worldwide. OSA has been established as an important risk factor for cardiovascular mortality and morbidity (Dobrosielski et al., 2017; de Vries et al., 2018; Lebkuchen et al., 2021). OSA contributes to cardiovascular diseases such as myocardial infraction (Porto et al., 2017), cardiac arrhythmias (May et al., 2017), coronary artery disease (Belaidi et al., 2016), and heart failure (Sanderson et al., 2021). Meanwhile, OSA is also associated with various conditions that increase the risk of cardiovascular diseases (CVD) themselves, such as hyperlipidemia (Gunduz et al., 2019) and atherosclerosis (Xue et al., 2017). Thus, a better understanding of OSA-associated cardiovascular disease is urgently needed, which will be helpful for improving the diagnosis and treatment of this disease.

Circular RNAs (circRNAs), a class of newly discovered noncoding RNAs, are reported to be extensively expressed across different species (Cai et al., 2019). Recently, emerging number of studies have focused on the function of circRNAs, indicating that circRNAs can modulate the function of certain miRNA by directly binding at its specific miRNA (Abbaszadeh-Goudarzi et al., 2020; A. ; Huang A. et al., 2020). Dysregulation of circRNAs has been found to be associated with multiple human diseases, including diabetes (Yang et al., 2020). For example, Zhao et al. reported that circRNAs were differentially expressed in type 2 diabetes mellitus (T2DM) and hsa_circ_0054633 may be served as a diagnostic biomarker of T2DM (Zhao et al., 2017). Furthermore, increasing studies have uncovered that some circRNAs exhibit a critical role in cancer development and progression. In non-small cell lung cancer, circNDUFB2 inhibits cancer progression and metastasis via the degradation of IGF2BPs (Li et al., 2021). To date, very little is known about the expression profile and potential role of circRNAs in OSA-induced cardiac injury.

In the current research, we first utilized circRNA microarray to investigate circRNA expression profiles in the mouse model of OSA-induced cardiac injury. Subsequently, we performed GO and KEGG enrichment analyses to annotate the biological functions of the differentially expressed circRNAs. To better understand the pathogenesis of OSA-induced cardiac injury, the functional circRNA-miRNA-mRNA regulatory modules were also constructed. Together, our findings indicated that circRNAs dysregulation may be associated with initiation and progression of OSA-induced cardiac injury and provide more potential biomarkers and new insights for OSA-related cardiovascular disease.

## Materials and Methods

### Animal

Male balb/c mice (17–21g, 6 weeks old) were supplied by Beijing Weitong Lihua Experimental Animal Technology Co., Ltd., Animal experiments were performed in accordance with the NIH Guide for Laboratory Animals. All mice were provided with standard mouse diet and tap water. The Experimental Animal Ethics Committee of the Second Affiliated Hospital of Fujian Medical University approved the animal protocol of this study.

### Myocardial CIH Injury Protocol

The animal model of CIH was established as previously described (Chen et al., 2019). Briefly, mice with CIH treatment were placed in the intermittent hypoxia system. The oxygen and nitrogen flow into the chamber was regulated by a gas control system. Ambient oxygen was servo-controlled to create an intermittent hypoxia condition. During a 2-min cycle, nitrogen was delivered to the chamber at a steady rate to reach 6% O_2_ for 60 s. After then, compressed air was pumped into the chamber to achieve 21% O_2_ for another 60 s. Mice were placed daily in the chamber for 30 cycles per h, 8 h/day for 7 days/week, for eight consecutive weeks. For the control group, mice were housed in the chamber with 21% O_2_ during the entire experiment. The oxygen concentration was calculated automatically with the help of an oxygen analyzer. All mice were euthanized at the end of CIH exposure. Their left ventricular tissues were collected.

### CircRNA Microarray Hybridization

A NanoDrop ND-1000 was utilized to quantify total RNA from each sample. Based on the Arraystar’s standard protocols, the sample preparation and microarray hybridization were conducted. In brief, we first digested total RNAs with Rnase R (Epicentre, Inc.) to eliminate linear RNAs and enrich circular RNAs. Then, with the use of a random priming method (Arraystar Super RNA Labeling Kit), the enriched circular RNAs were amplified and transcribed into fluorescent cRNA. Subsequently, the labeled cRNAs were then hybridized to the Arraystar Mouse circRNA Array V2. At last, the arrays were scanned by the use of the Agilent Scanner G2505C after having washed the slides.

### Microarray Data Analysis

The collected array images were analyzed by using Agilent Feature Extraction software (version 11.0.1.1). The R software limma package was further used by us for quantile normalization and subsequent data processing. CircRNAs exhibiting fold changes (FC) ≥1.5 and *p*-values ≤0.05 were considered statistically significant. Volcano plots was performed to show all differentially expressed circRNAs between the CIH and control groups. Fold change filtering was conducted to determine the differentially altered circRNAs between the two groups. Hierarchical clustering was also applied to display the distinguishable circRNA-expression patterns among the samples.

### Bioinformatic Analyses

Gene Ontology (GO) analysis was carried out to determine functional annotations of differentially expressed circRNAs and their target genes, which mainly included three independent ontologies (cellular component, molecular function and biological process). Moreover, Kyoto Encyclopedia of Genes and Genomes (KEGG) analysis was further performed to clarify the functions and interactions among these differentially expressed genes. In addition, Fisher’s exact test was performed to calculate the *p*-value. Data analysis was based on the KEGG database (https://www.genome.jp/kegg/) and GO database (http://geneontology.org). The *p*-value threshold was <0.05 and the count number >2. The top 10 enrichment GO entries and KEGG pathways of the dysregulated circRNAs were ranked and selected by enrichment score [-log10 (*p*-value)].

### Quantitative Real-Time Polymerase Chain Reaction (qRT-PCR)

qRT-PCR was conducted to verify the differential expression level of 10 selected circRNAs. We extracted the total RNA from cardiac tissues by using the TRIzol Reagent (Takara, Dalian, China). All primers were designed to span the distal ends of circRNAs using Primer 5 software (Table 1). We utilized the PrimeScript™ RT Reagent Kit (Takara, China) to synthesize cDNA. The qRT-PCR analyses were further carried out with the use of a TB Green™ Premix Ex Taq™ II (Takara, China) and an ABI Q2Real-time PCR system (Applied Biosystems, USA). *β*-actin was served as an internal control. Relative circRNAs expression was calculated by the 2^−ΔΔCt^ method.

**TABLE 1:** Primers used for qRT-PCR.

Genes	Forward and reverse sequence	Product length (bp)
β-actin	F:5′ GTA​CCA​CCA​TGT​ACC​CAG​GC3′	247
	R:5′AACGCAGCTCAGTAACAGTCC3′	
mmu_circRNA_006185	F:5′GTTACCACAAAGCAGAGAACACT 3′	65
	R:5′GCACATTCTTCATAACATCTGG 3′	
mmu_circRNA_014309	F:5′GTGAGAGACCTGAGAGGGATAG 3′	92
	R:5′TTCACTAACTTCCTTACGCTAATC 3′	
mmu_circRNA_014583	F:5′ TAT​AGC​GCC​AAG​GGA​AGC​A 3′	57
	R:5′ AGG​TCC​GGA​CAG​CTG​AGT​TG 3′	
mmu_circRNA_21856	F:5′CACTTTTTGGCTACTTTGTGCC 3′	99
	R:5′GTGAAGACACTCACGATGGGG 3′	
mmu_circRNA_26948	F:5′AGTATAGGCAGCTCTGGGATGA 3′	117
	R:5′ TGC​AAC​GAT​CAA​AGC​TGA​TG 3′	
mmu_circRNA_35821	F:5′ GCA​GAG​TCA​GAG​TTC​ACC​CAC 3′	85
	R:5′ CCC​TCA​CTT​ATT​TCC​TCC​AAA 3′	
mmu_circRNA_36076	F:5′ TTT​GTA​TTG​ACA​ACT​GGA​GCG 3′	103
	R:5′CCAAAGTCATAGACCATTGCCT 3′	
mmu_circRNA_23696	F:5′ CCT​AAG​TGC​CGT​ACC​AGC​T 3′	82
	R:5′ TGC​AGG​TTA​TTA​ATG​CCT​CAT 3′	
mmu_circRNA_43432	F:5′TCCATAAAGATTATTGAACTCTGA 3′	101
	R:5′GCCTCCTGTAGTGTTGTGAAA 3′	
mmu_circRNA_32974	F:5′ACAAAGAGGAGGAAGTCGGTC 3′	82
	R:5′ GGT​GAT​TTT​CAT​CGC​CAA​TAA​T 3′	

### Construction of a circRNA-miRNA-mRNA Regulatory Network

The two significant differentially expressed circRNAs were selected to build up a circRNA-miRNA-mRNA network by a software (Arraystar’s home-made miRNA target prediction software), which was based on miRanda and TargetScan. What’s more, the software that we used should consider the binding capacity of circRNA and microRNA, as well as the capacity and number of microRNA-mRNA binding sites. Finally, we visualized the established competing endogenous RNA (ceRNA) network by use of Cytoscape (Version 3.7.2).

### Statistical Methods

All data in this study were from at least three independent repeated experiments. Data were expressed as the means ± standard deviation. Student’s t-test was applied for comparisons between the CIH and control groups. Differences were deemed statistically significant at *p* < 0.05.

## Results

### Overview of CircRNA Expression

**FIGURE 1:**
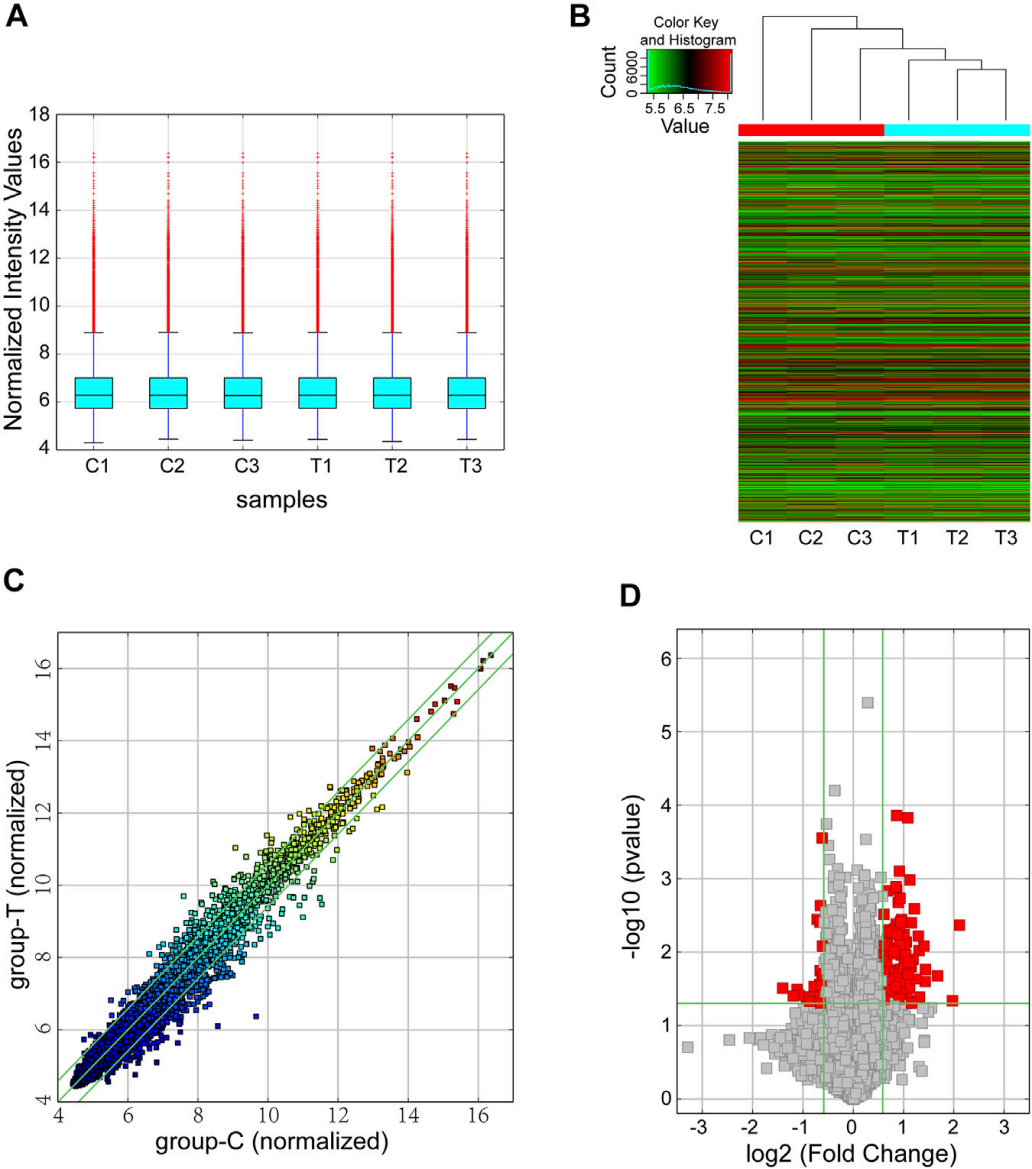
Overview of the microarray signatures. **(A)** The box plot was performed to show the profile distributions of the normalized intensities. **(B)** Hierarchical clustering revealed the differentially expressed circRNAs between the CIH groups and control tissues. **(C)** The scatter plot demonstrated the variation of the differentially expressed circRNAs between the two groups. The green lines represented 1.5-fold changes. circRNAs below or above the green lines indicated >1.5-fold downregulation or upregulation between the two compared groups. **(D)** Differentially expressed circRNAs were displayed by using volcano plot. The vertical green lines represented a 1.5-fold down or up, respectively. The horizontal green line exhibited a *p* value of 0.05 (-log10-scaled). The red squares indicated the dysregulated circRNAs with significant difference. CIH, chronic intermittent hypoxemia.

We performed circRNA microarray for screening dysregulated circRNAs with six samples (3 samples from the CIH group and 3 from the control group). With a threshold of FC ≥ 1.5 and *p* < 0.05, a total of 124 differentially expressed circRNAs were found in our mouse model of OSA-induced cardiac injury. Among them, 23 circRNAs were significantly downregulated while 101 were obviously upregulated. The box plot demonstrated that the circRNA median of the six samples was not different after quantile normalization (Figure 1A). Meanwhile, hierarchical clustering revealed a distinguishable circRNA expression profiling between CIH and control tissues (Figure 1B). Furthermore, scatter plot showed the variation of circRNA expression between groups (Figure 1C). Volcano plots was also used for visualizing differentially expressed circRNAs (Figure 1D).

**FIGURE 2:**
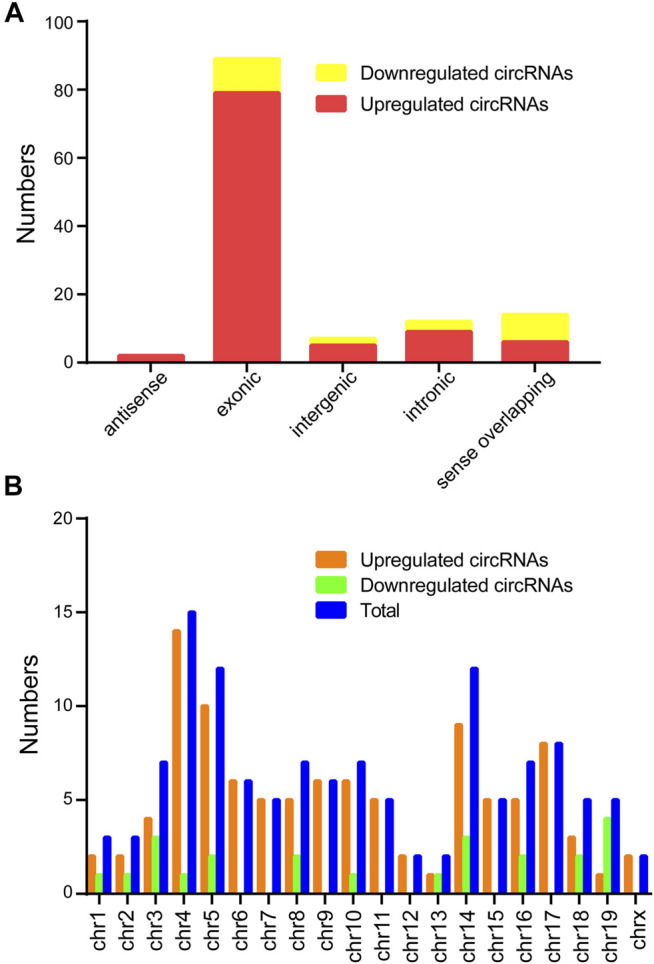
Classification and distribution of the differentially expressed circRNAs. **(A)** Classification of the altered expressed circRNAs was listed. **(B)** The number of altered expressed circRNAs was determined in each chromosome.

Classification of the dysregulated circRNAs was listed in the Figure 2A. The results found 101 upregulated circRNAs containing 79 exonic, 6 sense-overlapping, 9 intronic, 2 antisense, and 5 intergenic regions, whereas the 23 downregulated circRNAs comprised 8 sense-overlapping, 10 exonic, 2 intergenic, 0 antisense, and 3 intronic in CIH-tissue samples. In addition, chromosomal distribution analysis revealed that most circRNAs were located at chromosome 4, while few located at the X chromosome and chromosome 13 (Figure 2B).

### Validation of circRNA Expression

**FIGURE 3:**
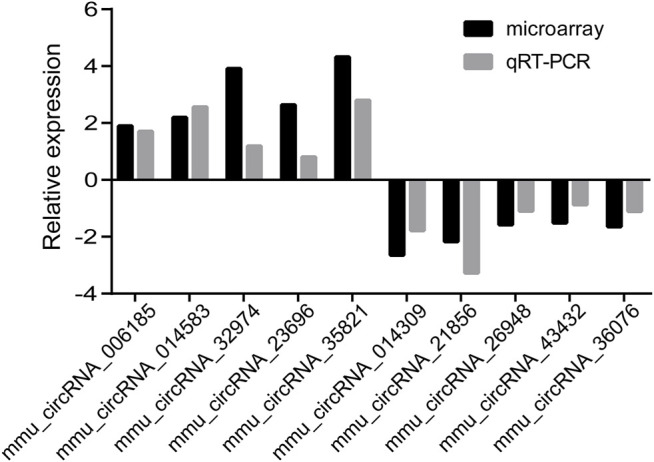
Relative fold changes of 10 randomly selected circRNAs by qRT-PCR and microarray. The downwards indicates downregulation, while upwards indicates upregulation.

To verify the microarray data, we randomly selected 10 dysregulated circRNAs from the microarray including 5 upregulated circRNAs (mmu_circRNA_006185, 014583, 32974, 23696, and 35821) and 5 downregulated circRNAs (mmu_circRNA_ 014309, 21856, 26948, 43432, and 36076) for further verification by qRT-PCR in samples. A general consistency was demonstrated between the microarray and qRT-PCR results. That is, four selected downregulated circRNAs and four selected upregulated circRNAs were validated (Figure 3). Our findings were in agreement with microarray results.

### GO and KEGG Analyses

**FIGURE 4:**
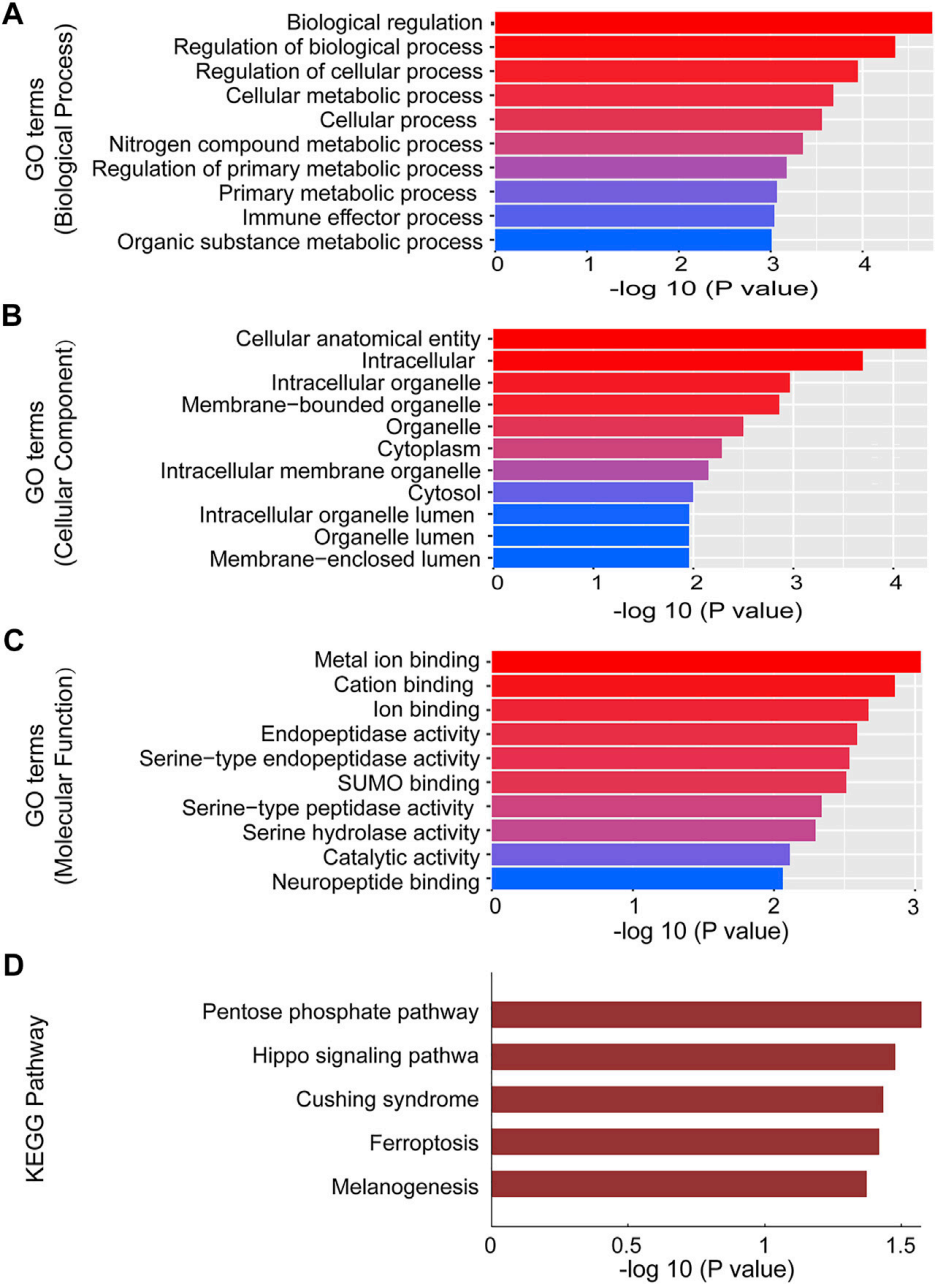
GO and Pathway analysis of mmu_circRNA_014309 and 21856. **(A)** Top 10 GO terms in BP. **(B)** Top 10 GO terms in CC. **(C)** Top 10 GO terms in MF. **(D)** Top 6 enrichment pathways. GO, Gene Ontology; CC, cellular components; BP, biological processes; MF, molecular functions.

To investigate the role of circRNAs on the expression of target genes, five circRNAs (2 of them were downregulated and 3 were upregulated) were further selected from the validated circRNAs to carry out GO and KEGG pathway analyses. GO analysis annotated genes targeted by the five altered expressed circRNAs, which included multiple biological processes, cellular components and molecular functions (Figures 4A–C). The most significant GO functions of mmu_circRNA_014309 and _21856 were related to intracellular organelle, metal ion binding, biological regulation, cation binding, regulation of biological process, intracellular, regulation of cellular process, cellular anatomical entity, cellular metabolic process, and nitrogen compound metabolic process. Additionally, KEGG pathway analysis suggested that numerous pathways involved in OSA-induced cardiac injury were associated with the dysregulated circRNAs. Our study found that enrichment of multiple key biological functions was engaged with OSA-induced cardiac damage. The results for mmu_circRNA_014309 and _21856 were listed in Figure 4D. The host genes of the altered expressed circRNAs were significantly associated with Pentose phosphate pathway, Cushing syndrome, the Hippo signaling pathway, Ferroptosis, and Melanogenesis.

### Construction an Interaction Network of ceRNA

**FIGURE 5:**
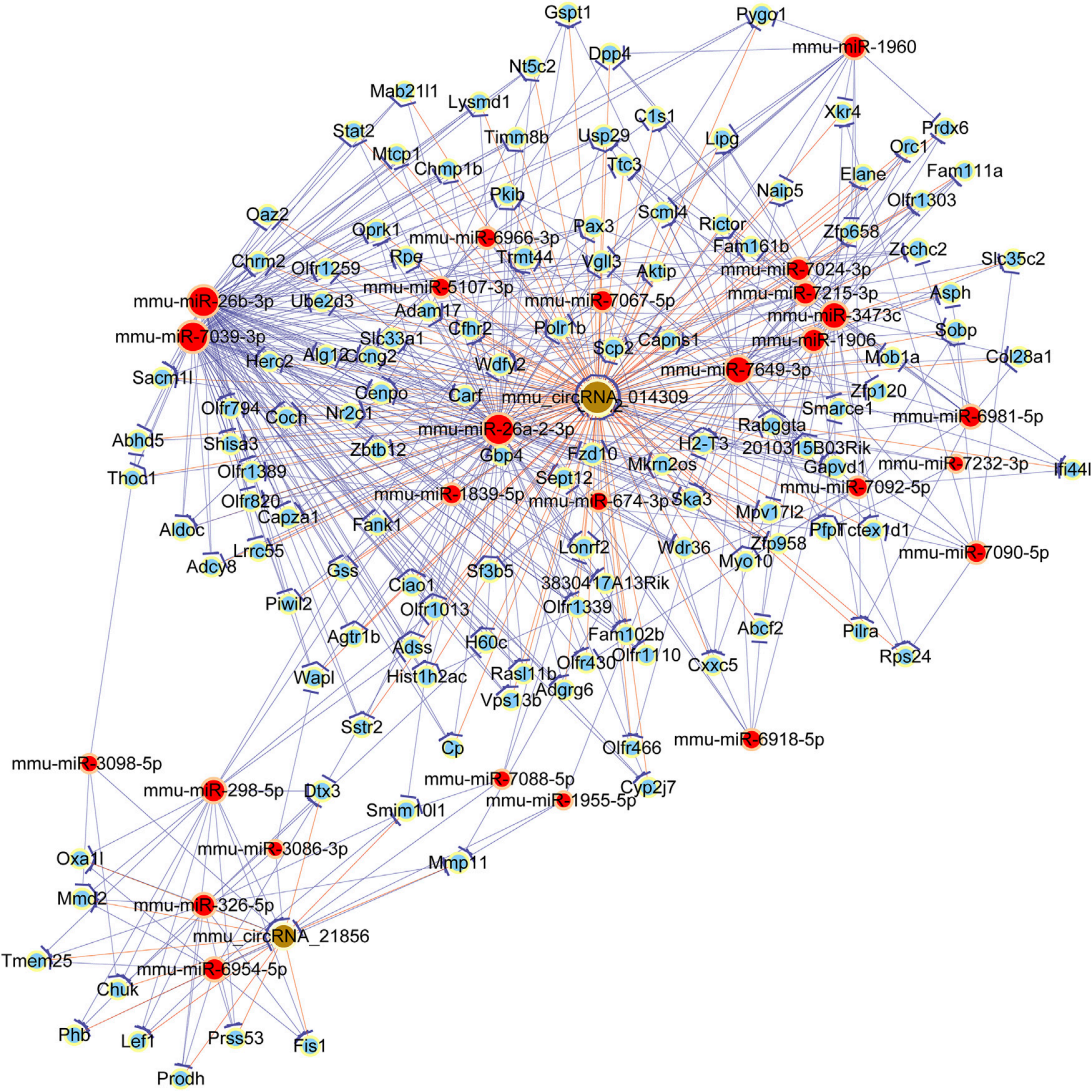
Establishment of circRNA-miRNA-mRNA network for two candidate circRNAs.

In order to explore the ceRNA network, the targets of dysregulated circRNAs and downstream genes were predicted by TargetScan and miRanda. In addition, to elucidate the bio-function of circRNAs took part in OSA-induced cardiac injury, we used Cytoscape software to construct a ceRNA network, based on the underlying effect of two selected circRNAs (mmu_circ_014309 and mmu_circ_21856), and their targeted miRNA and downstream mRNAs. The circRNA-miRNA-mRNA regulatory network was presented in Figure 5. The data indicated potential roles for the identified circRNAs as ceRNA ability of altering the expression of target genes. As shown in the picture, a total of 7 miRNAs (miR-326–5p, miR-298–5p, miR-3098–5p, miR-3086–3p, miR-6954–5p, miR-1955–5p, and miR-7088–5p) and corresponding target mRNAs were further predicted to have correlation with mmu_circRNA_21,856 in our current study. Meanwhile, a number of 20 miRNAs (mmu-miR-26b-3p, mmu-miR-26a-2-3p, mmu-miR-7039–3p, mmu-miR-7092–5p, mmu-miR-7067–5p, mmu-miR-7024–3p, mmu-miR-5107–3p, mmu-miR-6966–3p, mmu-miR-6918–5p, mmu-miR-7649–3p, mmu-miR-3473c, mmu-miR-1960, mmu-miR-7232–3p, mmu-miR-7090–5p, mmu-miR-1906, mmu-miR-1839–5p, mmu-miR-7092–5p, mmu-miR-6981–5p, mmu-miR-674–3p, and mmu-miR-7215–3p) and corresponding target mRNAs were further revealed to have correlation with mmu_circ_014309. Our finding provides us with innovative research strategy to uncover the underlying mechanism of mmu_circRNA_21,856 by revealing its associated miRNAs and investigating whether it can regulate its certain associated mRNAs expression.

## Discussion

To the best of our knowledge, this study is the first study reporting the specific circRNA expression profiles in the mouse model of OSA-induced cardiac injury. Additionally, we provide some potential targets and pathways of the circRNAs involved in OSA-induced cardiac damage. Our findings offer valuable clues to find the critical roles of circRNAs in the pathologic process of OSA-related cardiovascular disease.

As a novel heterogeneous class of ncRNAs, circRNAs have gained increased attention owing to their involvement with various forms of disease (Zhong et al., 2018). CircRNAs are abundant, endogenous, stable, conserved, and cell-type specific molecules, which could participate in regulating cell function (Kristensen et al., 2019). Increasing attention has focused on functions for circRNAs in human diseases, such as neurological diseases (W. Han et al., 2018; Chen et al., 2020a), cardiovascular disease (Zhang et al., 2020a; Tang et al., 2021), diabetes mellitus (Zhang et al., 2020b; Zaiou, 2020), and tumorigenesis (Goodall and Wickramasinghe, 2021; G. ; Huang et al., 2020b), especially cardiovascular diseases. For instance, Garikipati et al. found that circRNAs were altered expressed in the mouse model of post myocardial infarction (MI) (Garikipati et al., 2019). Furthermore, Ge et al. first revealed that a total of 185 circRNAs were significantly differentially expressed in the pathological process of ischemia/reperfusion (I/R) induced cardiac injury (Ge et al., 2019). Based on the high-throughput circRNA microarray data, we also found 101 upregulated circRNAs and 23 downregulated circRNAs in the CIH group compared with the control group. However, their roles in OSA-induced cardiac injury remain largely unknown.

To investigate the possible roles of circRNAs on OSA-induced cardiac injury, 124 differentially expressed circRNAs were identified. qRT-PCR of the 10 differentially expressed circRNAs confirmed that 8 of them were statistically significant and concordant with microarray assay results (*p* < 0.05). The main reasons for these discrepancies are: Firstly, owing to the high cost of circRNA microarray chips, a limited number of samples were performed for screening of circRNAs. Secondly, the microarray chip technology is highly dependent on computational analyses, there is a certain false positive rate. Thirdly, different results are likely to occur due to the inherent differences among mice, such as the degree of pathological alterations. Finally, the difference detected between the two groups may be due to the small sample size used for verification by qRT-PCR.

To unveil new insight into the potential roles of the dysregulated circRNAs in the development of OSA-induced cardiac damage, both GO and pathway analysis were further performed to predict the biological functions and underlying mechanisms of the targeted genes. The most important GO functions were associated with cellular metabolic process, regulation of biological process, biological regulation, cellular anatomical entity, and regulation of cellular process. Meanwhile, to gain more credible biological functions, KEGG pathway analysis was also carried out to identify the significant pathways. It was found that the dysregulated transcripts were related to the Hippo signaling pathway, Cushing syndrome, and Ferroptosis. Furthermore, Chen et al. demonstrated that the Ferroptosis pathway was involved in the animal model of CIH-induced liver injury (Chen et al., 2021; L. D. ; Chen et al., 2020b). Therefore, it was indicated that the Ferroptosis pathway might play an important role in the development of OSA-induced injury. Further investigations are still in urgent need to confirm these findings.

In recent years, a large number of circRNAs have been discovered. Mounting evidence has shown that circRNAs have emerged as a novel special class of endogenous noncoding RNAs. To date, more and more studies have reported strong associations between circRNAs and cardiovascular disease (Li et al., 2020; Zhang and Wang, 2020; Yu et al., 2021). What is more, circRNAs can regulate parent gene expression or function as miRNA sponges to affect disease initiation and progression. For example, the silence of circHIPK3 could ameliorate MI-induced cardiac dysfunction *via* targeting miR-93–5p and inhibiting the Rac1/PI3K/Akt pathway (Wu et al., 2021). Recently, more studies demonstrated that circRNAs can function mainly as miRNA sponges (Misir et al., 2020). In our study, we first found many differentially expressed circRNAs in the heart tissue after CIH exposure. Meanwhile, we also predicted a putative circRNA/microRNA/mRNA interaction with the use of miRanda and TargetScan software. To our interest, we revealed that mmu_circRNA_21856 can tightly bind to miR-326–5p, which may be a potential sponge of miR-326–5p. Meanwhile, Li et al. reported that miR-326–5p could enhance the angiogenic ability of endothelial progenitor cells (EPCs) and promote functional cardiac repair of EPCs through targeting Wnt1 in an acute MI model (Li et al., 2019). mmu_circRNA_21,856 is likely to represent a novel mediator of OSA-induced cardiac injury. Therefore, we propose a hypothesis that circRNA_21,856 may act as an efficient sponge of miR-326–5p and then influence the downstream mRNAs expression. However, due to the limited available data on functions of miRNAs and circRNAs, more circRNA/microRNA interaction should be further investigated in the future research.

The present study was the first to characterize the circRNA expression profile in the process of OSA-associated cardiovascular diseases. However, several limitations should be acknowledged in this study. Firstly, due to the relatively small sample size the generalizability of the present results is difficult to establish. Secondly, the exact mechanisms of these candidate circRNAs in OSA-induced cardiac injury pathogenesis were not explored in our study. Thus, further studies will be required to investigate the function and regulatory mechanisms of these dysregulated circRNAs. Thirdly, although mice exhibit a high degree in sequence homology with humans, whether these results can also be successfully applied to humans needs further confirmation. Last, a well performed characterization of nullified and/or down-regulated mmu_circ_014,309 and mmu_circ_21856 circRNA mouse, would greatly improve the quality of the manuscript.

In conclusion, our study has provided the first evidence of differentially-expressed circRNAs in the occurrence and development of OSA-induced cardiac injury. We also have made preliminary predictions about the potential functions of these circRNAs by bioinformatics analysis and a ceRNA network. These findings may yield new insight into the underlying mechanisms of OSA-induced cardiac damage, and might present novel molecular targets for treatment of OSA-related cardiovascular disease.

## Data Availability

The raw data supporting the conclusions of this article will be made available by the authors, without undue reservation.
